# Seasonal and Regional Dynamics of Mastitis Pathogens: Insights From a 3-Year Study in China

**DOI:** 10.1155/tbed/3631905

**Published:** 2025-11-26

**Authors:** Yawei Qiu, Shaodong Fu, Naiyan Sun, Bo Yang, Shiyuan Feng, Jinqiu Zhang, Dancheng Yang, Jinfeng Miao

**Affiliations:** ^1^MOE Joint International Research Laboratory of Animal Health and Food Safety, College of Veterinary Medicine, Nanjing Agricultural University, Nanjing 210095, Jiangsu, China; ^2^Sanya Research Institute, Nanjing Agricultural University, Sanya 572024, Hainan, China; ^3^College of Life Sciences, Nanjing Agricultural University, Nanjing 210095, Jiangsu, China

**Keywords:** contagious mastitis, cow, environmental mastitis, pathogens

## Abstract

Mastitis is one of the biggest problems and an economic burden facing the dairy industry with a strong negative impact on animal welfare, productivity, and food safety. This study conducted a systematic investigation and analysis of the epidemiological characteristics of mastitis in selected regions of China (significant region for raw milk production). By collecting an extensive number of milk samples from clinical mastitis cases and utilizing methodologies such as bacterial isolation, genomic DNA extraction, and pathogen identification, the research elucidated the distribution patterns and trends of pathogenic bacteria responsible for mastitis across various regions and seasons. A total of 7177 milk samples were analyzed, identifying pathogenic bacteria in 3720 samples, which corresponds to a detection rate of 51.83%. Sixteen species of pathogenic bacteria were identified, with *Klebsiella*, coagulase-negative staphylococci (CoNS), and *Escherichia coli* (*E. coli*) being the predominant pathogens, representing 21.33%, 20.63%, and 18.72%, respectively. The study revealed significant seasonal and regional variations in the prevalence of pathogenic bacteria associated with mastitis. Detection rates of these bacteria were significantly higher in samples collected from May to September compared to other months, with September showing the highest detection rate at 85.94%. Furthermore, the southern region of China demonstrated the highest detection rate of pathogenic bacteria, with a prevalence of 94.98%. This study explored the pathogenicity and antimicrobial resistance profiles of the predominant bacterial strains, and proposed targeted prevention and control strategies based on these insights. The overarching aim is to provide a scientific basis for the effective management of mastitis, thereby alleviating the economic impact on the dairy farming industry.

## 1. Introduction

Mastitis is a significant disease that affects the health and productivity of dairy cow globally and poses a major economic burden to the dairy industry in China. It is estimated that this disease results in annual economic losses exceeding 20 billion yuan in China, primarily due to reduced milk yield, increased veterinary costs, and milk discard [1]. Beyond the economic impact, mastitis also raises public health concerns as pathogens, toxins, and drug residues in milk can enter the food chain, potentially compromising consumer safety.

Mastitis can be triggered by various pathogens, and its epidemiology is broadly categorized into two types: contagious and environmental [2]. Contagious mastitis is typically caused by pathogens such as *Staphylococcus aureus* (*S. aureus*), *Streptococcus agalactiae* (*S. agalactiae*), and *Corynebacterium bovis* (*C. bovis*), which spread directly between cows or via contaminated equipments. In contrast, environmental mastitis is associated with opportunistic pathogens present in the environment, including *Escherichia coli* (*E. coli*), *Klebsiella spp*., *Streptococcus dysgalactiae* (*S. dysgalactiae*), and nonhemolytic *Staphylococcus spp*. These environmental pathogens often thrive in suboptimal housing conditions, such as damp stalls or overcrowded barns, and their prevalence can fluctuate with seasonal changes.

According to the latest global monitoring, *S. aureus*, *Streptococcus aenolactis*, *E. coli*, and *Klebsiella pneumoniae*(*K. pneumoniae*)have replaced traditional dominant bacteria, accounting for 75.3% of clinical cases of bovine mastitis worldwide [3]. During the same period, a retrospective analysis of 319,000 milk samples from the University of California, Davis between 2009 and 2023 showed that environmental pathogens such as nonaureus staphylococci and coliforms have replaced traditional infectious bacteria as the main cause of cow mastitis, and showed significant seasonal differences in winter and summer [4]. Numerous studies have been conducted on the etiology of mastitis across various regions in China, revealing a diverse array of pathogen profiles. He et al. [5] identified several pathogenic bacteria in their study of mastitis, with *E. coli* and *S. aureus* being the most prevalent, comprising 32.1% and 28.7% of isolates, respectively. Coagulase-negative staphylococci (CoNS) also showed a significant presence at 21.3%, while *Streptococcus spp*. and *Klebsiella spp*. accounted for 8.6% and 4.2%, respectively. Although pathogen distribution varied regionally, *E. coli* and *S. aureus* were consistently the primary causes of mastitis in China [5]. An epidemiological survey in Inner Mongolia from 2015–2024 confirmed these findings, highlighting *E. coli* and *S. aureus* as the leading pathogens. Additionally, pathogens such as *Enterobacter spp*., *Salmonella spp*., and *Pasteurella spp*. were detected [6]. Wang et al. [8] examined the prevalence of *S. aureus* linked to mastitis in China from 2000 to 2020, finding a total detection rate of 36.23%. The rates in Northern and Southern China were 36.56% and 35.75%, respectively [7]. Chen et al. [7] studied clinical mastitis prevalence and risk factors in dairy cows across mainland China from 1982–2022, reporting an overall prevalence of 10%. Key pathogens identified include *E. coli*, *K. pneumoniae*, *S. aureus*, CoNS, *S. agalactiae*, *S. uberis*, and other streptococcal species [8].

Understanding the epidemiological patterns of mastitis is crucial for developing effective prevention and control strategies. Previous studies have highlighted the role of seasonal variations and regional differences in the incidence and pathogen profiles of mastitis. For instance, some regions experience higher mastitis rates in summer due to elevated temperatures and humidity, which favor pathogen proliferation, while others report increased cases in spring or autumn. Similarly, variations in management practices, climate conditions, and cow genetics across different regions can influence the prevalence and types of mastitis-causing pathogens.

This study aims to provide a comprehensive epidemiological investigation of mastitis in specific regions of China. By analyzing a large number of milk samples collected over an extended period, we seek to identify the predominant pathogens, their seasonal and regional distribution patterns, and their pathogenic and drug-resistance characteristics. The findings of this research will contribute to a better understanding of the disease dynamics and offer science-based insights for optimizing mastitis control measures in the dairy industry.

## 2. Materials and Methods

### 2.1. Sample Source

This study collected 7177 milk samples from large-scale dairy farms in 22 regions—including Inner Mongolia, Anhui, Hebei, Jiangsu, Ningxia, Heilongjiang, and Shandon, among others—between April 2019 and October 2022 to investigate the prevalence of clinical mastitis (Table 1).

All farm veterinarians and personnel participating in this investigation underwent formal training and adhered rigorously to established protocols for the collection of milk samples. Throughout the study, the Nanjing Agricultural University International Reference Laboratory for the Diagnosis of *Streptococcus suis* offered assistance in analyzing milk samples obtained from dairy cows afflicted with mastitis.

### 2.2. Sample Collection

Farm veterinarians diagnose mastitis in cows by assessing changes in the udders and milk. Clinical mastitis manifests as redness, swelling in the udders, enlarged mammary lymph nodes, varying udder quarter temperatures, abnormal milk color (gray or light yellow), presence of lumps or flocs, and bleeding in milk leading to a red appearance. Subclinical mastitis is typically diagnosed by ranch veterinarians using the California mastitis test (CMT). This test involves the reaction of milk with a commercial CMT reagent, which leads to the destruction of somatic cells in the milk, releasing DNA. Subsequent reactions result in milk precipitation or gel formation. The quantity of somatic cells correlates with the amount of precipitation or gel produced, indirectly indicating the presence of mastitis and the extent of inflammation.

Collectors follow a specific protocol when collecting milk samples. Initially, operators wear disposable gloves and cleanse the udder with a disinfected warm towel, followed by an iodine bath. Subsequently, the udder is dried, and the first three streams of milk from each teat are discarded before milk collection commences from the proximal teats. Milk from each teat is collected into a 50 mL sterile tube. Upon completion of sample collection, the sampling bottle is sealed, labeled with the cow number, udder quarter, and sampling date, and transported to the laboratory for analysis within 24 h. Furthermore, a paper sampling form specifying the ranch name and sampling time is to be attached. The serial number on the sampling form corresponds to the cow number.

All samples were collected over a 43-month period from April 2019 to October 2022, with monthly sampling conducted. Samples were slightly fewer in March 2020, August 2021, and February 2022 due to pandemic-related site closures, but the overall monthly distribution difference was <5%, which did not affect seasonal trend assessment.

### 2.3. Microbiological Culture and Identification

#### 2.3.1. Microbiological Isolation and Purification

In this investigation, we employed the standard protocols outlined by the National Mastitis Council (NMC) for the isolation of pathogenic microorganisms from milk samples. A sterile cotton swab was utilized to sample the milk by dipping it into the milk bottle, removing excess liquid, and streaking it onto MacConkey agar and blood agar plates. Subsequently, the plates were inverted and placed in an incubator set at 37°C for 18–36 h. For further analysis, a sterile 1.5 mL EP tube was prepared by adding 1 mL of THB medium followed by 100 *μ*L of serum. A single colony exhibiting typical morphology was selected and transferred into the EP tube, which was then subjected to incubation on a shaker at a constant temperature of 37°C for 18–36 h. All procedures involving isolation and purification were conducted within a laminar flow hood.

#### 2.3.2. DNA Extraction and Identification

DNA extraction using the water bath method involves selecting the strain isolated on a nutrient agar slant with an inoculating loop and transferring it into 50 *μ*L of solution. Subsequently, the sample is subjected to heat treatment in a constant-temperature water bath set at 80°C for 15 min. Upon completion of the DNA extraction process, the extracted DNA should be stored at −20°C. Each batch shall include a positive control (ATCC 25922) and a negative control (nuclease-free water).

PCR amplification: The DNA extracted by the water bath method was subjected to PCR amplification using the 16S PCR universal primers purchased from Sangon Biotech (Shanghai) Co., Ltd.

  27F：5′-AGAGTTTGATCCTGGCTCAG-3′
  1492R：5′-GGTACCTTGTTACGACTT-3′

The total reaction volume was 50 *μ*L, comprising 25 *μ*L of Taq PCR Mastermix, 2 *μ*L of forward primer (27F), 2 *μ*L of reverse primer (1492R), 2 *μ*L of DNA, and 19 *μ*L of ddH_2_O. Nucleic acid-free water was then added to bring the total volume to 50 *μ*L.

Reaction conditions: Predenaturation: 94°C, 4 min, with a cycle number of 1; PCR amplification: denaturation at 94°C for 30 s, annealing at 57°C for 30 s, extension at 72°C for 90s, and repeating the cycle 30 times; final extension: 72°C, 10 min, with a cycle number of 1; incubation: 10°C, ∞.

Sequencing result alignment: Perform a BLAST alignment of the sequenced sequences on NCBI to obtain sequences with high similarity to the sequenced ones, thus completing the identification of the isolated and purified strains.

### 2.4. Result Judgment and Statistical Analysis

Positive determination as bacterial dissociation positive or 16S rRNA sequencing similarity ≥99%. Statistical summaries were conducted using Microsoft Excel 2020 on the bacteria isolated and purified from the samples, establishing a database that includes regions, years, pathogen types, and quantities.

## 3. Results

### 3.1. Culture Results

In this study, 7177 milk samples were collected, with 3720 testing positive for pathogens and 3457 testing negative. Among the 3720 positive samples, 16 different pathogenic bacteria were identified, including 188 *S. aureus*, 710 CoNS, 125 *S. agalactiae*, 63 *S. dysgalactiae*, 143 *Streptococcus uberis*, 97 other *Streptococcus spp*., 734 *Klebsiella spp*., 644 *E. coli*, 186 *Enterobacter spp*., 35 *Cryptobacter pyogenes*, 44 *Pseudomonas aeruginosa*, 76 unidentified pathogens, 1 *Salmonella spp*., 21 *Pasteurella spp*., 260 *Lactococcus spp*., 121 *Enterococci* strains, and 718 strains of other bacteria.

### 3.2. Relationship Between Detection Rate of Pathogenic Bacteria in Milk Samples and Seasonal Differences

**Figure 1:**
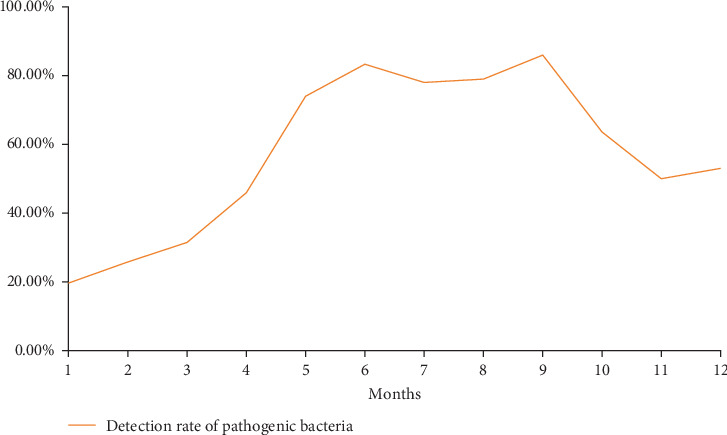
Trend of pathogen detection rates across different months.

Significant monthly variations in pathogen detection rates were observed (Table 2). From January to May, as temperatures rose, the detection rate of pathogenic bacteria increased markedly (Figure 1), with January recording the lowest rate at 19.65%. During the summer months, from May to September, the detection rates were consistently higher than in other months, with minimal intermonth differences, surpassing the overall sample average. Notably, September had the peak detection rate of 85.94%. From October to December, a decline in detection rates was observed (Figure 1), although they remained above the overall average. In October, the rate was 63.57%, slightly exceeding the rates in November and December. The detection rate of samples was significantly lower in spring and winter compared to summer and autumn. Pathogen types also varied by month. July recorded the highest diversity, with 16 species of pathogenic bacteria, while spring and autumn followed closely with 13–15 species. January had the fewest, with only nine species detected.

### 3.3. Relationship Between Pathogen Detection Rate in Milk Samples and Regional Variability

Regional variation in the prevalence of clinical mastitis pathogens was noted, although species differences were not significant (Table 3). South China had the highest detection rate of milk-related pathogens at 94.98%, followed by Northwest and Southwest China at 91.41% and 89.84%, respectively. In contrast, North and East China showed lower detection rates of 48.97% and 50.56%.

**Figure 2:**
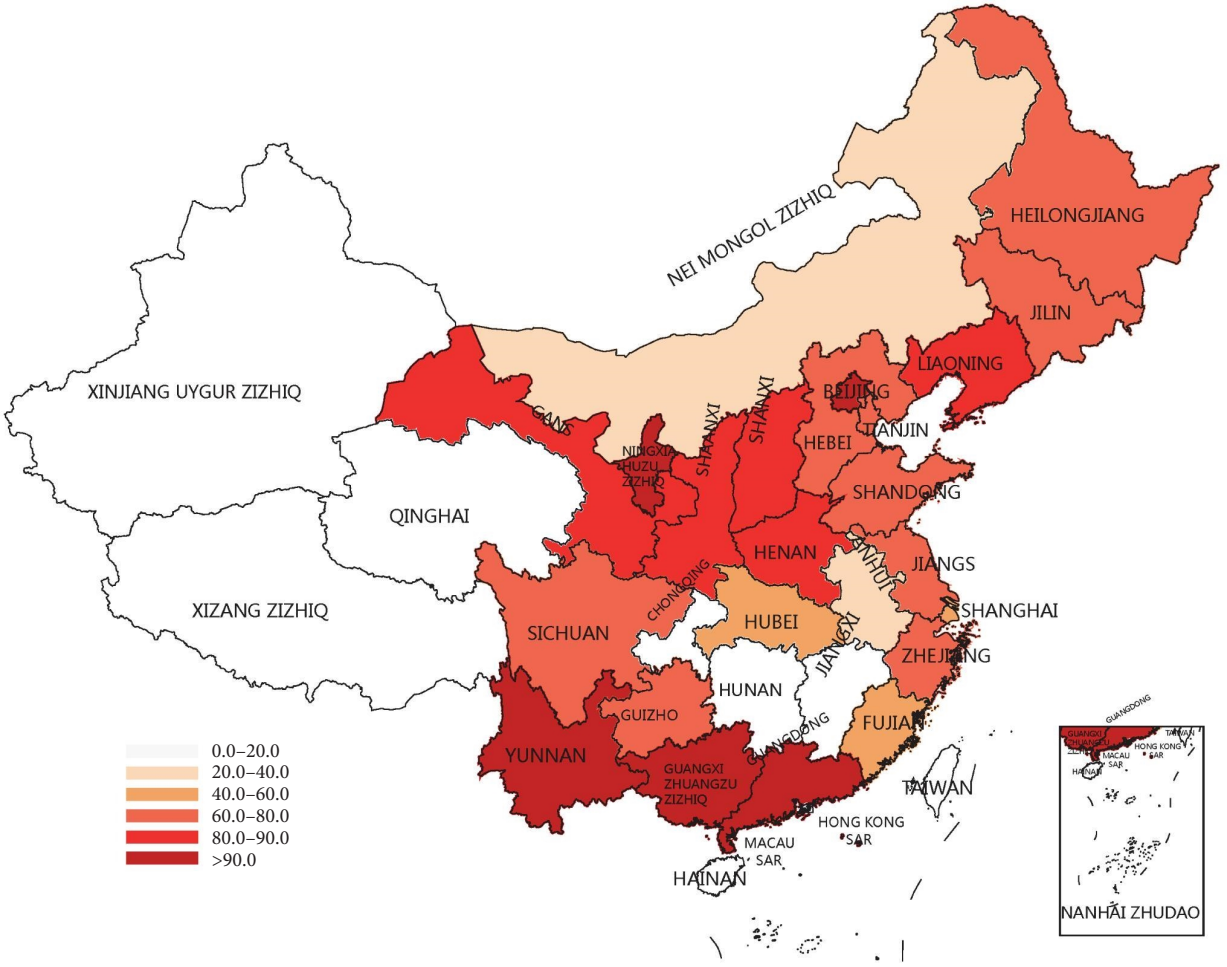
Heat map of the detection rate of milk-like pathogens in some provinces of China.

Regions such as Yunnan Province, Beijing City, Guangxi Zhuang Autonomous Region, Guangdong Province, and Ningxia Hui Autonomous Region exhibit high milk-sample pathogen detection rates, all exceeding 90% (Figure 2). Conversely, Fujian Province, Shanghai City, Hubei Province, Inner Mongolia Autonomous Region, and Anhui Province report rates below 50%. Notably, Anhui Province has the lowest detection rate among all regions, at just 30.75%.

### 3.4. Detection of Different Pathogens Causing Bovine Mastitis in Some Regions of China

Among the 3720 samples tested positive in this study, the pathogenic bacteria with the highest detection rate were *Klebsiella spp*. with 17.62%, followed by CoNS, *E. coli*, *Lactococcus lactis*, *Enterobacteriaceae*, *S. aureus*, and *Streptococcus lactis*, with detection rates of 17.04%, 15.46%, 6.24%, 4.46%, 4.32%, and 3.43%. Some common mastitis pathogens such as *Streptococcus spp*. (*S.agalactiae*, *Streptococcus pyogenes*, other *Streptococci*), *Cryptobacterium septicum*, *Pseudomonas aeruginosa*, *Aerococcus glabrata*, *Salmonella spp*., *Pasteurella spp*., and *Enterococcus spp*. were also detected in this study at a rate of about 14%.

#### 3.4.1. Relationship Between Detection Rates of Different Pathogens and Seasonal Variability

The predominant pathogens causing clinical mastitis showed both similarities and differences monthly (Tables 4 and 5). From March to October, the three most frequently detected organisms were CoNS, *Klebsiella spp*., and *E. coli*. CoNS, consistently infectious year-round, peaked at a 65.52% detection rate in February. *Klebsiella spp*. had its lowest detection rates in February and November, ranking among the top three in all other months, with a peak of 30.70% in July. *E. coli* reached its highest detection rate of 24.85% in August, consistently exceeding 14.88%. *S. aureus* was most prevalent in November, with a detection rate of 25.30%.

The seasonal distribution of bacterial strains in the samples revealed distinct patterns. In spring, *Klebsiella spp*., *E.coli*, and CoNS were the most prevalent, each comprising 20.77% and 18.82%, respectively. During summer, *Klebsiella spp*. increased to 25.85%, with *E. coli* at 20.77% and CoNS at 17.64%. In fall and winter, the most frequently detected strains were *Klebsiella spp*., *E. coli*, CoNS, *S. aureus*, and *Enterobacteriaceae*, in that order.

#### 3.4.2. Relationship Between the Detection Rate of Different Pathogenic Bacteria and Regional Variability

The dominant pathogens causing clinical mastitis vary by region in China (Table 6). In Northeast, North, East, and Central China, *Klebsiella spp*., *E.coli*, and CoNS are prevalent, collectively accounting for approximately 50% of pathogenic detections. *Klebsiella spp*. detection rates were 23.78%, 15.10%, 20.67%, and 12.30% in these regions, respectively. *E. coli* was detected at 14.92%, 18.35%, 9.35%, and 12.30%, while CoNS were found at 21.21%, 16.27%, 20.84%, and 19.25%. In North China, *Lactococci spp*. were identified in 7.69% of cases. In South China, *Klebsiella spp*., *S. aureus*, and CoNS had the highest detection rates at 26.92%, 15.87%, and 8.65%, respectively, followed by *E. coli* and *S. agalactiae* at 6.25% and 5.77%. In Northwest China, *E. coli*, *Lactococcus spp*., and CoNS were most frequently detected at 24.15%, 13.78%, and 13.13%, respectively, with *Klebsiella spp*. also notable at 11.18%. In Southwest China, *Klebsiella spp*., *E. coli*, and *S. agalactiae* were most prevalent, with detection rates of 21.74%, 18.26%, and 9.57%, respectively.

## 4. Discussion

### 4.1. Relationship Between the Incidence of Clinical Mastitis in Dairy Cows and Seasonal Variations

The incidence of dairy cow mastitis exhibits pronounced seasonal variations. In a large-scale ranch in Xinjiang, the highest mastitis rate was observed in January (6.03%), while the lowest occurred in April (1.27%). These differences in mastitis incidence across seasons and months were statistically significant [9]. The elevated incidence during the summer months can be attributed to the combined effects of environmental factors. Specifically, the rising temperatures, increasing humidity, and stuffy conditions often lead to a decline in the physical condition of dairy cows. Additionally, poor sanitation, inadequate ventilation, wet bedding, high stocking density, and limited exercise space in the cowsheds create an environment conducive to the rapid proliferation and transmission of pathogenic bacteria, thereby resulting in a relatively high mastitis prevalence during this season. In addition, it is worth noting that in Northern China, the high-incidence period of mastitis usually occurs in spring, while in Southern China, this problem tends to occur in summer. However, in the Yunnan-Guizhou region in Southwestern China, due to the relatively stable annual temperature changes, there is no significant seasonal difference in the incidence of mastitis. This is consistent with the findings of Zhang et al. [10] The possible reason is that during the seasonal alternation, the drastic temperature fluctuations put stress on dairy cows, and at the same time, the ranch staff failed to provide appropriate care in a timely manner, thus creating opportunities for pathogen invasion.

### 4.2. Relationship Between the Detection Rate of Clinical Mastitis Samples in Dairy Cows and Seasonal and Regional Variations

This study's analysis of clinical mastitis samples from dairy cows in various Chinese regions revealed that detection rates of pathogenic bacteria were significantly elevated from May through September compared to other months. The detection rates during these 5 months were consistently higher than the overall rate, with September exhibiting the highest detection rate. July saw the greatest diversity of pathogenic bacteria, identifying 16 species. This pattern likely correlates with rising national temperatures during these months, which increase the complexity of pathogenic bacteria in pastures. Concurrently, pasture management and hygiene may not improve sufficiently, leading to a higher incidence of clinical mastitis in dairy cows, alongside increased detection rates and bacterial diversity. From October onward, as temperatures decrease, both the detection rate and bacterial diversity decline, reaching their lowest levels in January.

The detection rates of clinical mastitis samples were significantly lower in North China, East China, Central China, and Northeast China compared to Southwest China, Northwest China, and South China. This disparity may be attributed to more effective pasture management and improved sanitation conditions in the former regions, leading to milder clinical symptoms and easier treatment of mastitis in dairy cows. Additionally, the relatively high annual average temperatures in South and Southwest China could be a contributing factor to the elevated detection rates observed in these regions.

### 4.3. Relationship Between Dominant Pathogens of Clinical Mastitis and Seasonal/Regional Variations

Mastitis is caused by a diverse array of pathogenic bacteria, each exhibiting distinct pathogenic mechanisms, epidemic trends, and drug resistance profiles, posing significant challenges for dairy farm management. Analysis of seasonal detection rates revealed that CoNS consistently maintained high prevalence year-round, emerging as the primary pathogen responsible for clinical mastitis nationwide. *E. coli* and *Klebsiella spp*. also showed substantial detection rates throughout the year, with *E. coli* peaking in August and *Klebsiella spp*. in July. Conversely, *S. aureus* exhibited marked seasonal variation, predominating in autumn and winter. This seasonal pattern is similarly observed in dairy farms in Northwestern Ethiopia and Northeastern India, where autumn and winter conditions favor its survival and transmission [11, 12].

Regional variations exist in the predominant pathogenic bacteria causing clinical mastitis. In Northeast, North, East, and Central China, environmental pathogens like *Klebsiella spp*., *E. coli*, and CoNS are most common. Conversely, *S. aureus* is more prevalent in South China. The findings of this study in South China align with those of Ma et al. [13] and Song et al. [14], who identified *S. aureus* as the leading pathogen in mastitis in this region, with a significantly higher detection rate than in other parts of China. We recommend that ranches in South China adopt targeted measures to address the prevalence of dominant pathogenic bacteria. Cows infected with *S. aureus* should be isolated to prevent contact with healthy cows, thereby limiting bacterial spread. Prompt culling should be considered when necessary. Regular cleaning of cowsheds is essential to minimize pollution from feces and sewage, providing cows with a clean and dry environment to reduce teat exposure to pathogens. Nutritional supplementation with vitamin E, selenium, vitamin A, and *β*-carotene, along with balanced trace elements like copper and zinc, can enhance immunity and reduce susceptibility to *S. aureus*. For ranches in Northeast, North, East, and Central China, effective environmental management is crucial. This includes maintaining dry and clean cowsheds, ensuring proper layout to prevent overcrowding, implementing a scientific disinfection protocol, and regularly monitoring cattle health.

### 4.4. Analysis of Dominant Pathogenic Bacteria Causing Clinical Mastitis in Dairy Cows in Some Regions of China

In many regions of China, environmental pathogens are now the primary cause of clinical mastitis in dairy cows [15]. This study identified *Klebsiella spp*., CoNS, and *E. coli* as the predominant flora in the sampled area, comprising 21.33%, 20.63%, and 18.72% of the total samples, respectively.

CoNS, typically considered opportunistic pathogens, are linked to environmental factors. Ubiquitous in nature and found on human and animal skin and mucosa, they spread via direct contact, aerosols, or contaminated equipment and environments. In a study, dairy cows with mastitis in large-scale farms in Hohhot, Inner Mongolia, predominantly harbored CoNS, aligning with our findings [16]. These bacteria can infiltrate dairy cow mammary glands through contaminated environments and milking equipment, colonizing the teat canal and mammary tissues, leading to infections. Such infections result in decreased milk production and shortened peak lactation periods. As treatment primarily involves antibiotics, these pathogens often exhibit high antibiotic resistance and can develop multidrug resistance. Thus, for farms with high incidences of these bacteria, we recommend thorough environmental disinfection, prevention of cross-contamination, regular health monitoring of cattle, reduced antibiotic use, and the strategic use of immune-enhancing products to bolster dairy cow resistance.

Research indicates that *E. coli* and *Klebsiella spp*. are the predominant environmental pathogens, aligning with our findings on dairy cow mastitis in certain regions of China [17]. *Klebsiella*-induced clinical mastitis often correlates with prolonged subclinical mastitis, inadequate inflammatory response, and poor prognosis [18]. This accounts for the observed seasonal variations in *Klebsiella spp*. and *E. coli* prevalence. Further data reveal that *E. coli*-related mastitis typically manifests acutely, with an effective immune response and brief symptoms [19]. Domestic studies report a high *Klebsiella spp*. detection rate in wood chip and sawdust bedding, highlighting the advantages of fresh sand bedding in controlling mastitis [20]. Thus, farms with high *Klebsiella* prevalence might benefit from switching to sand bedding and enhancing hygiene.

## 5. Conclusions

This study investigated the epidemiological characteristics of mastitis in certain Chinese regions and found significant seasonal and geographical differences in its incidence. The pathogen detection rate and variety were highest from May to September, with the most detected pathogens in July, which is attributed to the summer heat and humidity that facilitate pathogen proliferation and transmission, as well as suboptimal cowshed sanitation and husbandry conditions that lower cows' resistance. In terms of geography, the North China, East China, Central China, and Northeast China regions had relatively low sample detection rates, possibly due to better farm management and hygiene; in contrast, the South China and Southwest China regions had high detection rates, which might be linked to their consistently high annual temperatures. In terms of pathogen distribution, CoNS had the highest detection rate throughout the year and were the primary pathogens causing clinical mastitis nationwide. *E. coli* and *Klebsiella spp*. also had high detection rates, with regional variations in dominant pathogens. In view of this, it is recommended that farms implement targeted prevention and control measures based on their specific circumstances, such as improving the environment, disinfection, nutritional supplementation, and disease monitoring, to reduce the incidence of mastitis, minimize losses, and promote the healthy development of the dairy cow farming industry.

This study presents the first systematic 3-year spatiotemporal mapping of mastitis pathogens in China's primary milk production regions, analyzing 7177 milk samples. The research quantitatively correlates regional climate-management variations with pathogen prevalence, offering evidence-based guidance for developing seasonal disinfection, bedding replacement, and regional medication strategies.

In this study, there were significant regional differences in the sample size. The confidence intervals of the detection rates in regions with small samples were relatively wide, so caution should be exercised when extrapolating. From 2020 to 2022, the sampling volume decreased due to the lockdown of farms during the pandemic, which might affect the seasonal estimation in extreme months. In addition, confounding variables such as parity, lactation stage, and feeding mode were not included. Whether the seasonal/regional differences have independent effects still needs to be verified by a multivariate model. Finally, there is a lack of systematic data on virulence genes and drug resistance, and further research is needed on the pathogenicity and treatment difficulty of highly detected strains.

## Figures and Tables

**Table 1:** Specific source information of mastitis milk samples from selected regions in China, 2019–2022.

Selected regions	Number of samples				Total
	2019	2020	2021	2022	
Anhui	91	64	796	57	1008
Beijing	–	–	–	24	24
Fujian	–	–	–	65	65
Gansu	7	–	–	14	21
Guangdong	49	32	35	15	131
Guangxi	–	29	35	24	88
Guizhou	–	–	16	–	16
Hebei	180	126	87	503	896
Henan	48	13	–	101	162
Heilongjiang	81	55	174	173	483
Hubei	78	40	16	–	134
Jilin	–	–	83	18	101
Jiangsu	145	117	122	99	483
Liaoning	–	–	–	20	20
Inner Mongolia	285	190	1255	205	1935
Ningxia	21	24	55	516	616
Shandong	26	65	177	64	332
Shanxi	10	4	11	11	36
Shaanxi	30	–	–	8	38
Shanghai	–	57	54	–	111
Sichuan	–	50	–	–	50
Tianjin	29	–	–	29	58
Yunnan	–	27	13	22	62
Zhejiang	12	109	145	41	307

*Note:* The difference in sample size is mainly affected by the number of local cow stocks and the outbreak of mastitis: Inner Mongolia and Hebei are national milk source bases with a large stock, so there are many samples; Liaoning, Shanghai, and other places have only a small number of cooperative pastures, and the sample is ≤40.

**Table 2:** The results of the detection of pathogens in different seasons.

Project	Spring			Summer			Autumn			Winter			Total
	Mar	Apr	May	Jun	Jul	Aug	Sept	Oct	Nov	Dec	Jan	Feb	
Number of samples	900	1228	653	790	613	760	576	280	188	149	229	811	7177
Number of pathogenic bacteria detected in the samples	283	564	483	658	478	600	495	178	94	79	45	209	4166
Pathogen detection rate (%)	31.44	45.93	73.97	83.29	77.98	78.95	85.94	63.57	50.00	53.02	19.65	25.77	—
Number of pathogenic bacteria detected	15	15	14	15	16	15	15	14	13	12	9	14	—

*Note:* Seasonal differences in pathogen detection rates were statistically significant (*χ^2^* = 287.4, *p*  < 0.001). The overall detection rate in summer (May–September) ranged from 78% to 86%, significantly higher than that in winter (19%–26%), with Bonferroni correction showing *p*  < 0.001). July recorded 16 species, marking the annual peak.

**Table 3:** The results of detection of bacterial pathogens in different regions of China.

Region	Northeast China	North China	East China	South China	Central China	Northwest China	Southwest China
Total number of samples	604	2949	2306	219	296	675	128
Number of samples with detected pathogens	429	1444	1166	208	187	617	115
Pathogen detection rate (%)	71.03	48.97	50.56	94.98	63.18	91.41	89.84
Number of detected pathogenic species	14	15	15	14	16	14	14

*Note:* The detection rate of pathogens showed significant differences between regions (*χ^2^* = 412.7, *p*  < 0.001). The detection rate in South China was as high as 94.98%, significantly higher than that in North China (48.97%), East China (50.56%) and Northeast China (71.03%) (Bonferroni correction *p*  < 0.001).

**Table 4:** The results of clinical mastitis detection of various pathogens in different months (spring and summer).

Pathogen	Spring						Summer					
	Mar		Apr		May		Jun		Jul		Aug	
	Number	Detection rate (%)	Number	Detection rate (%)	Number	Detection rate (%)	Number	Detection rate (%)	Number	Detection rate (%)	Number	Detection rate (%)
*S. aureus*	10	4.42	6	1.35	20	5.10	21	3.69	21	5.04	16	3.13
CoNS	36	15.93	85	19.06	76	19.39	119	20.91	44	10.55	101	19.77
*S. agalactiae*	24	10.62	10	2.24	—	—	28	4.92	27	6.47	7	1.37
*S. dysgalactiae*	6	2.65	7	1.57	8	2.04	7	1.23	4	0.96	8	1.57
*S. uberis*	11	4.87	31	6.95	12	3.06	6	1.05	17	4.08	17	3.33
Other *Streptococcus spp*.	1	0.44	19	4.26	10	2.55	10	1.76	18	4.32	12	2.35
*Klebsiella spp*.	36	15.93	89	19.96	96	24.49	123	21.62	128	30.70	136	26.61
*E. coli*	51	22.57	85	19.06	85	21.68	104	18.28	80	19.18	127	24.85
*Enterobacter spp*.	7	3.10	23	5.16	17	4.34	20	3.51	15	3.60	27	5.28
*Trueperella pyogenes*	1	0.44	4	0.90	4	1.02	3	0.53	8	1.92	2	0.39
*Pseudomonas aeruginosa*	2	0.88	2	0.45	7	1.79	9	1.58	6	1.44	14	2.74
*Aerococcus viridans*	16	7.08	5	1.12	6	1.53	11	1.93	6	1.44	10	1.96
*Salmonella spp*.	—	—	—	—	—	—	—	—	1	0.24	—	—
*Pasteurella spp*.	3	1.33	3	0.67	4	1.02	2	0.35	4	0.96	3	0.59
*Lactococcus spp*.	15	6.64	64	14.35	31	7.91	88	15.47	25	6.00	12	2.35
*Enterococcus spp*.	7	3.10	13	2.91	16	4.08	18	3.16	13	3.12	19	3.72

**Table 5:** The results of clinical mastitis detection of various pathogens in different months (autumn and winter).

Pathogen	Autumn						Winter					
	Sept		Oct		Nov		Dec		Jan		Feb	
	Number	Detection rate (%)	Number	Detection rate (%)	Number	Detection rate (%)	Number	Detection rate (%)	Number	Detection rate (%)	Number	Detection rate (%)
*S. aureus*	32	8.12	12	9.92	21	25.30	9	14.06	1	2.27	11	6.32
CoNS	83	21.07	20	16.53	14	16.87	5	7.81	13	29.55	114	65.52
*S. agalactiae*	14	3.55	1	0.83	2	2.41	9	14.06	—	—	3	1.72
*S. dysgalactiae*	9	2.28	2	1.65	3	3.61	3	4.69	1	2.27	5	2.87
*S. uberis*	19	4.82	6	4.96	9	10.84	7	10.94	2	4.55	6	3.45
Other *Streptococcus spp*.	13	3.30	5	4.13	2	2.41	2	3.13	4	9.09	1	0.57
*Klebsiella spp*.	64	16.24	30	24.79	7	8.43	9	14.06	13	29.55	3	1.72
*E. coli*	64	16.24	18	14.88	13	15.66	3	4.69	5	11.36	9	5.17
*Enterobacter spp*.	64	16.24	7	5.79	2	2.41	2	3.13	2	4.55	—	—
*Trueperella pyogenes*	1	0.25	2	1.65	1	1.20	7	10.94	—	—	2	1.15
*Pseudomonas aeruginosa*	3	0.76	1	0.83	—	—	—	—	—	—	1	0.57
*Aerococcus viridans*	4	1.02	3	2.48	1	1.20	1	1.56	3	6.82	10	5.75
*Salmonella spp*.	—	—	—	—	—	—	—	—	—	—	—	—
*Pasteurella spp*.	1	0.25	—	—	—	—	—	—	—	—	1	0.57
*Lactococcus spp*.	10	2.54	4	3.31	5	6.02	—	—	—	—	6	3.45
*Enterococcus spp*.	13	3.30	10	8.26	3	3.61	7	10.94	—	—	2	1.15

**Table 6:** The results of detection of clinical mastitis in different regions of China.

Pathogen	Northeast China		North China		East China		South China		Central China		Northwest China		Southwest China	
	Number	Detection rate (%)	Number	Detection rate (%)	Number	Detection rate (%)	Number	Detection rate (%)	Number	Detection rate (%)	Number	Detection rate (%)	Number	Detection rate (%)
*S. aureus*	24	5.59	46	3.19	59	5.06	33	15.87	3	1.60	14	2.27	1	0.87
CoNS	91	21.21	235	16.27	243	20.84	18	8.65	36	19.25	81	13.13	6	5.22
*S. agalactiae*	2	0.47	30	2.08	46	3.95	12	5.77	3	1.60	21	3.40	11	9.57
*S. dysgalactiae*	6	1.40	18	1.25	23	1.97	1	0.48	2	1.07	5	0.81	8	6.96
*S. uberis*	21	4.90	45	3.12	47	4.03	8	3.85	15	8.02	4	0.65	3	2.61
Other *Streptococcus spp*.	4	0.93	28	1.94	35	3.00	6	2.88	8	4.28	14	2.27	2	1.74
*Klebsiella spp*.	102	23.78	218	15.10	241	20.67	56	26.92	23	12.30	69	11.18	25	21.74
*E. coli*	64	14.92	265	18.35	109	9.35	13	6.25	23	12.30	149	24.15	21	18.26
*Enterobacter spp*.	16	3.73	52	3.60	62	5.32	8	3.85	12	6.42	30	4.86	6	5.22
*Trueperella pyogenes*	7	1.63	12	0.83	11	0.94	—	—	1	0.53	—	—	4	3.48
*Pseudomonas aeruginosa*	—	—	8	0.55	12	1.03	8	3.85	12	6.42	5	0.81	—	—
*Aerococcus viridans*	7	1.63	34	2.35	23	1.97	3	1.44	5	2.67	4	0.65	—	—
*Salmonella spp*.	—	—	—	—	—	—	—	—	1	0.53	—	—	—	—
*Pasteurella spp*.	2	0.47	12	0.83	1	0.09	1	0.48	1	0.53	3	0.49	1	0.87
*Lactococcus spp*.	11	2.56	111	7.69	41	3.52	1	0.48	9	4.81	85	13.78	2	1.74
*Enterococcus spp*.	10	2.33	47	3.25	27	2.32	2	0.96	8	4.28	22	3.57	5	4.35

## Data Availability

The data that support the findings of this study are available from the corresponding author upon reasonable request.
